# A novel small-molecule selective activator of homomeric GIRK4 channels

**DOI:** 10.1016/j.jbc.2022.102009

**Published:** 2022-05-04

**Authors:** Meng Cui, Keman Xu, Kirin D. Gada, Boris Shalomov, Michelle Ban, Giasemi C. Eptaminitaki, Takeharu Kawano, Leigh D. Plant, Nathan Dascal, Diomedes E. Logothetis

**Affiliations:** 1 Department of Pharmaceutical Sciences, School of Pharmacy, Bouvé College of Health Sciences, Northeastern University, Boston, Massachusetts, USA; 2 Center for Drug Discovery, Northeastern University, Boston, Massachusetts, USA; 3 Department of Physiology and Pharmacology and Sagol School of Neuroscience, School of Medicine, Tel Aviv University, Tel Aviv, Israel; 4 Chemistry and Chemical Biology, College of Science, Northeastern University, Boston, Massachusetts, USA

**Keywords:** Kir3 channels, drug–channel interaction, molecular docking, molecular dynamics simulations, mutagenesis, electrophysiology, ACh, acetylcholine, Ba, barium, CIBN, cryptochrome-interacting basic helix–loop–helix N-terminal fragment, CRY2, cryptochrome 2, GIRK, G protein–sensitive inwardly rectifying potassium channel, HAC15, human adrenocortical 15 cell line, HBC, helix bundle crossing, 3hi2one-G4, 3-[2-(3,4-dimethoxyphenyl)-2-oxoethyl]-3-hydroxy-1-(1-naphthylmethyl)-1,3-dihydro-2H-indol-2-one, HK, high potassium, I_KACh_, acetylcholine-activated inwardly rectifying K^+^ current, IVM, ivermectin, LK, low potassium, PA, primary aldosteronism, PIP2, phosphatidylinositol-4,5-bisphosphate, PMC, pro-opiomelanocortin, S-HLX, slide helix, TEVC, two-electrode voltage clamp, TM, transmembrane domain, TIRF, total internal reflection fluorescence, VMN, ventromedial nucleus

## Abstract

G protein–sensitive inwardly rectifying potassium (GIRK) channels are important pharmaceutical targets for neuronal, cardiac, and endocrine diseases. Although a number of GIRK channel modulators have been discovered in recent years, most lack selectivity. GIRK channels function as either homomeric (*i.e.*, GIRK2 and GIRK4) or heteromeric (*e.g.*, GIRK1/2, GIRK1/4, and GIRK2/3) tetramers. Activators, such as ML297, ivermectin, and GAT1508, have been shown to activate heteromeric GIRK1/2 channels better than GIRK1/4 channels with varying degrees of selectivity but not homomeric GIRK2 and GIRK4 channels. In addition, VU0529331 was discovered as the first homomeric GIRK channel activator, but it shows weak selectivity for GIRK2 over GIRK4 (or G4) homomeric channels. Here, we report the first highly selective small-molecule activator targeting GIRK4 homomeric channels, 3hi2one-G4 (3-[2-(3,4-dimethoxyphenyl)-2-oxoethyl]-3-hydroxy-1-(1-naphthylmethyl)-1,3-dihydro-2H-indol-2-one). We show that 3hi2one-G4 does not activate GIRK2, GIRK1/2, or GIRK1/4 channels. Using molecular modeling, mutagenesis, and electrophysiology, we analyzed the binding site of 3hi2one-G4 formed by the transmembrane 1, transmembrane 2, and slide helix regions of the GIRK4 channel, near the phosphatidylinositol-4,5-bisphosphate binding site, and show that it causes channel activation by strengthening channel–phosphatidylinositol-4,5-bisphosphate interactions. We also identify slide helix residue L77 in GIRK4, corresponding to residue I82 in GIRK2, as a major determinant of isoform-specific selectivity. We propose that 3hi2one-G4 could serve as a useful pharmaceutical probe in studying GIRK4 channel function and may also be pursued in drug optimization studies to tackle GIRK4-related diseases such as primary aldosteronism and late-onset obesity.

G protein–gated inwardly rectifying potassium (GIRK) channels mediate the inhibitory effects of various neurotransmitters acting through G protein–coupled receptors and G proteins present in excitable cells, such as in the heart and nervous system (1). Four GIRK channel family members (GIRK1–GIRK4) have been identified, but each is expressed either in homotetramers (GIRK2 and GIRK4) or in heterotetramers with the nonfunctional homomeric subunits (GIRK1 and GIRK3) (1). The GIRK1 subunit, although inactive by itself, enhances heteromeric channel activity when associated with either GIRK4 or GIRK2 subunits. The cardiac GIRK channel (I_KACh_) is a heterotetramer composed of GIRK1 and GIRK4 subunits expressed in the atria and pacemaking cells, and its activation slows heart rate (1). GIRK4 (or G4) homotetramers have also been reported to be expressed in atrial cells (2), where vagally released acetylcholine (ACh) activates the ACh-regulated potassium current, I_KACh_ (3).

It has been demonstrated that atrial fibrillation cannot be induced in GIRK4 knockout mice, suggesting the critical involvement of this current in atrial fibrillation (3). Thus, GIRK1/4 inhibitors could act as potentially promising antiarrhythmic agents. Although GIRK4 is not abundantly expressed in the brain (4), expression has also been detected in various neuronal populations, particularly in the pro-opiomelanocortin (POMC) and the ventromedial nucleus (VMN) neurons of the hypothalamus (5). Studies found that GIRK4 knockout mice were predisposed to late-onset obesity, by exhibiting greater food intake and a decrease in energy expenditure compared with wildtype mice. Similarly, inhibition of POMC and VMN neurons reduces energy expenditure and promotes food intake (5). Because GIRK4 channels reside in the POMC and VMN neurons, it is likely that GIRK4 is linked to changes in metabolic function and reduced satiety, leading to obesity.

In addition to the heart and brain, GIRK4 homomeric channels are also expressed in the adrenal cortex. Multiple mutations of GIRK4 channel cause primary aldosteronism (PA), which is a disease characterized by hypersecretion of aldosterone. PA is the most common cause of secondary hypertension and accounts for approximately 10% of patients with newly diagnosed hypertension (6). Loss of selectivity mutations, such as GIRK4 G151R, T158A, and L168R, which are located at or near the selectivity filter, were reported to yield K^+^/Na^+^ nonselective GIRK4 and GIRK1/4 channels (7). GIRK4 mutations R52H, E246K, and G247R, which are located in the cytosolic N-terminal and C-terminal domains, resulted in a loss-of-function phenotype (8). Therefore, GIRK4 activators could serve as a potential treatment for patients with PA.

GIRK channels are gated by the Gβɣ dimer of GTP-binding proteins (9), which bind the cytoplasmic domain between the DE and LM loops (Fig. S1) of two adjacent subunits (10, 11). Another intracellular channel activator is Na^+^, which binds within the CD loop (Fig. S1) of GIRK2 and GIRK4 that possess a critical Asp residue (12, 13, 14, 15), which coordinates Na^+^. Gβγ allosterically regulates interactions of the channel with the lipid molecule phosphatidylinositol-4,5-bisphosphate (PIP_2_) (12, 16, 17). Na^+^ has also been found to work by increasing the affinity of the channel to PIP_2_ (12, 13, 14). Direct interactions of PIP_2_ with the channel stabilize the GIRK channel gates, the HBC (helix bundle crossing), and the cytosolic G loop, in the open state (18, 19). The simultaneous presence of Gβɣ/Na^+^ shows synergism for channel activation *via* PIP_2_ (20) as Gβɣ controls predominantly the HBC gate, whereas Na^+^ controls the cytosolic G-loop gate (17).

Several activators of GIRK channels have been reported, such as alcohols, the bioflavonoid naringin, and the urea-scaffold compounds ML297 and GAT1508, all of which have been found to directly activate GIRK channels in a G protein–independent but PIP_2_-dependent manner (21, 22, 23, 24). Inhibitors on the other hand can act as pore blocking, such as the potent small bee venom peptide, tertiapin, which competes with the activator naringin for a similar site (25), or as allosteric inhibitors, such as the volatile anesthetics (*e.g.*, halothane), by interfering with the mechanism of Gβɣ activation (22, 26). Yet with the exception of GAT1508, which shows complete specificity for GIRK1/2 heterotetramers, none of the other pharmacological regulators target a single GIRK heteromeric or homomeric channel. A specific activator of a GIRK4-containing subunit in the central nervous system could be desirable for modulating late-onset obesity, whereas in the periphery, an activator would be desirable for PA and an inhibitor for reducing AF.

Here, we report a novel selective activator, 3hi2one-G4 (3-[2-(3,4-dimethoxyphenyl)-2-oxoethyl]-3-hydroxy-1-(1-naphthylmethyl)-1,3-dihydro-2H-indol-2-one), that achieves the desired specificity for GIRK4 homomeric channel over other GIRK homotetramers or heterotetramers. The binding site of 3hi2one-G4 in the GIRK4 channel was validated and characterized. 3hi2one-G4 could serve as a useful pharmaceutical probe to study GIRK4 channel function as well as toward drug discovery for diseases such as PA and late-onset obesity.

## Results

### 3hi2one-G4 activates GIRK4 homomeric channels

**Figure 1:**
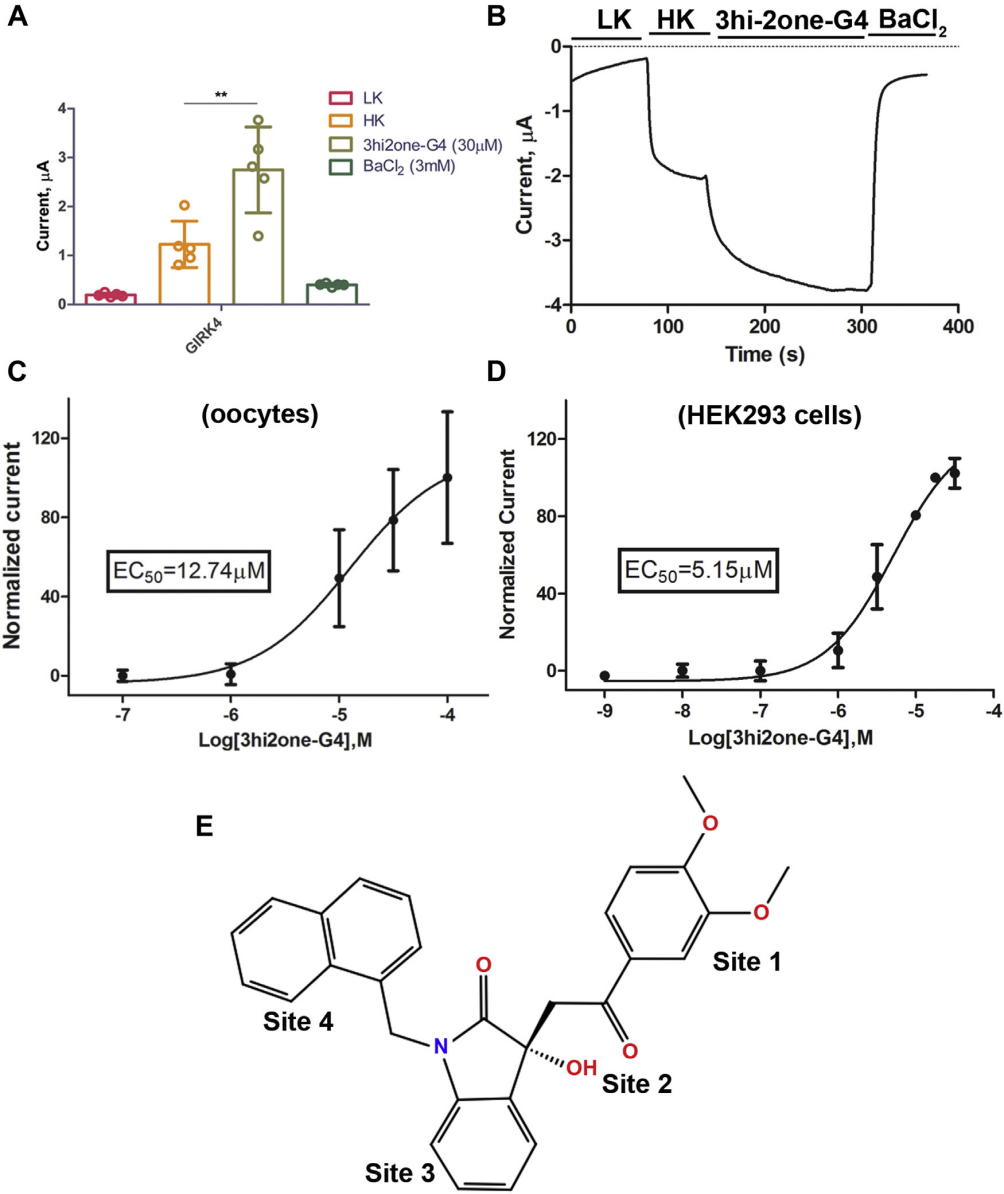
**3hi2one-G4 activates GIRK4 homomeric channel.***A*, GIRK4 channel is activated by 3hi2one-G4 (30 μM) (TEVC). The *asterisks* indicate significant differences tested by unpaired Student's *t* test (∗∗*p* < 0.01) (data are mean ± SD, N = 5). *B*, representative traces of responses to the 3hi2one-G4 compound (30 μM) of GIRK4 channel. *C*, dose–response curves of the 3hi2one-G4 compound activation on the GIRK4 channel (EC_50_ = 12.74 μM) from TEVC recordings of *Xenopus* oocytes expressing GIRK4 channels (N = 5). *D*, dose–response curves of the 3h2one-G4 compound activation on the GIRK4 channel (EC_50_ = 5.15 μM) from patch-clamp recordings of HEK293 cells expressing GIRK4 channels (N > 5) (data are mean ± SD). *E*, the chemical structure of 3hi2one-G4. GIRK, G protein–sensitive inwardly rectifying potassium channel; HEK293, human embryonic kidney 293 cell; 3hi2one, 3-[2-(3,4-dimethoxyphenyl)-2-oxoethyl]-3-hydroxy-1-(1-naphthylmethyl)-1,3-dihydro-2H-indol-2-one; HK, high potassium solution ND96K; LK, low potassium solution ND96; TEVC, two-electrode voltage clamp.

The 3hi2one-G4 compound (Fig. 1*E*) was discovered through virtual high-throughput screening of the Specs database (www.specs.net) searching for modulators of the homomeric GIRK4 channel. Using two-electrode voltage clamp (TEVC) and patch-clamp experiments, we found that 3hi2one-G4 activates GIRK4 channels expressed both in *Xenopus* oocytes and human embryonic kidney 293T cells. Figure 1 shows that 3hi2one-G4 activates heterologously expressed GIRK4 homomeric channels at a concentration of 30 μM. 3hi2one-G4 activated GIRK4 whole-cell currents in a dose-dependent manner, with an EC_50_ of 12.74 μM in TEVC experiments in *Xenopus* oocytes (Fig. 1*C*) and 5.15 μM in whole-cell patch-clamp experiments in human embryonic kidney 293 cells, respectively (Fig. 1*D*). The maximum concentration of 3hi2one-G4 tested was 100 μM because of its limited solubility.

### 3hi2one-G4 selectively activates GIRK4 over other GIRK channels

**Figure 2:**
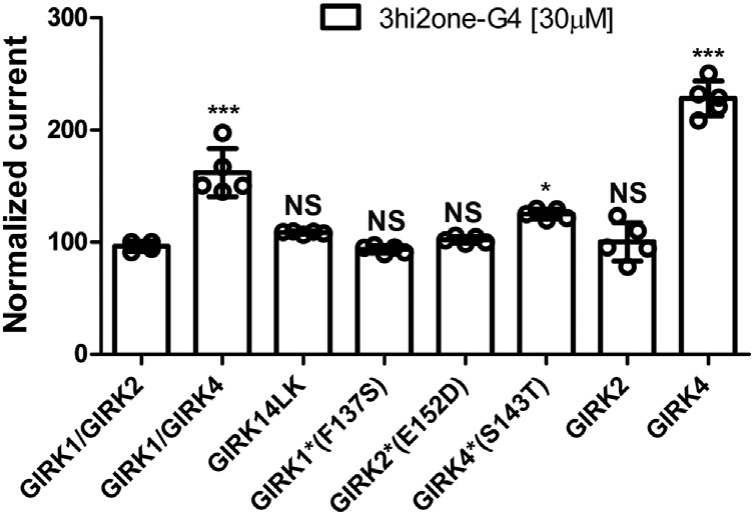
**3hi2one-G4 selectively activates GIRK4 channels.** TEVC recordings of GIRK1/2, GIRK1/4, GIRK14LK, GIRK1∗(GIRK1:F137S), GIRK2∗(GIRK2:E152D), GIRK4∗(GIRK4:S143T), GIRK2, and GIRK4 channels expressed in *Xenopus* oocytes. The basal current (normalized) is measured in a high potassium (HK) solution ND96K. The *asterisks* indicate significant differences tested by one-way ANOVA Tukey's test (∗∗∗*p* < 0.001, ∗*p* < 0.05) compared with GIRK1/2 (data are mean ± SD, N ≥ 4). GIRK, G protein–sensitive inwardly rectifying potassium channel; 3hi2one, 3-[2-(3,4-dimethoxyphenyl)-2-oxoethyl]-3-hydroxy-1-(1-naphthylmethyl)-1,3-dihydro-2H-indol-2-one; TEVC, two-electrode voltage clamp.

The selectivity of 3hi2one-G4 was tested on GIRK1/2, GIRK1/4, GIRK14LK (linked version of GIRK1 and GIRK4), GIRK1∗(GIRK1:F137S), GIRK2∗(GIRK2:E152D) (27), GIRK4∗(GIRK4:S143T) (4), GIRK2, and GIRK4 channels. The GIRK1∗, GIRK2∗, and GIRK4∗ channels were included in our studies since they produce relatively larger currents than their corresponding wildtype GIRK homomeric channels and are widely used for GIRK homomeric channel function studies in the field. Figure 2 shows that 3hi2one-G4 activated GIRK4, GIRK4∗, and GIRK1/4 channels, but it did not activate GIRK1∗, GIRK2∗, GIRK2, and GIRK1/2 channels. Since the activation seen upon coexpression of GIRK1 and GIRK4 subunits could have resulted from formation of GIRK4 homomeric channel, we used an in-tandem linked construct, GIRK14LK, to eliminate possible effects on GIRK4 homomers, as this construct produces only heteromeric channels. 3hi2one-G4 at 30 μM failed to activate GIRK14LK heteromeric channel, suggesting that the GIRK1/4 activation was likely because of activation of GIRK4 homotetramers. Thus, these results indicate 3hi2one-G4 to be a highly selective activator of GIRK4 homomeric channels.

### 3hi2one-G4 enhances GIRK4 channel interactions with PIP_2_

**Figure 3:**
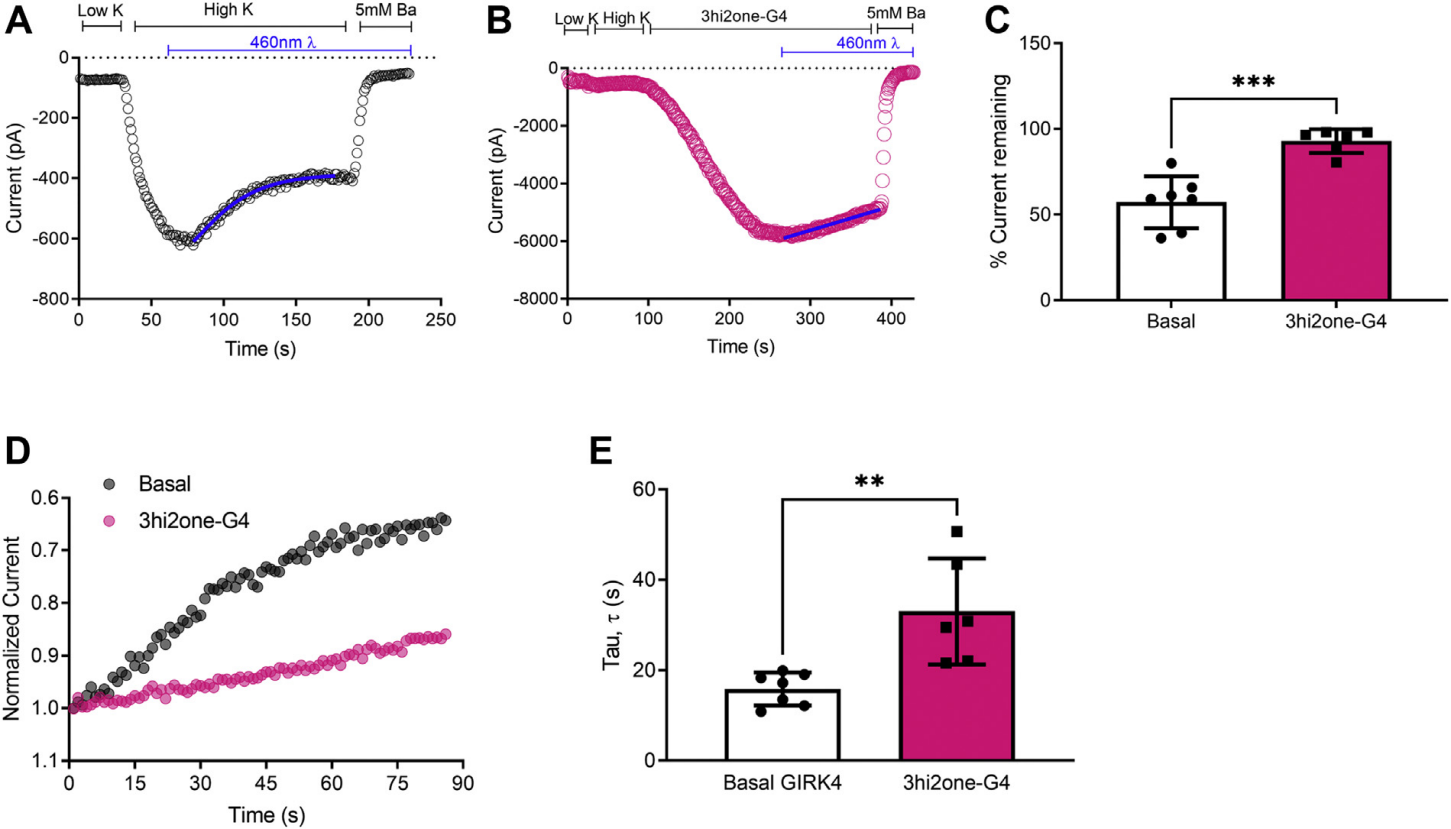
**3hi2one-G4 decreases GIRK4 current inhibition after PIP**_**2**_**dephosphorylation by 5-ptaseOCRL in HEK cells.***A*, representative plot of GIRK4 current decrease (control, *black open circle*). *B*, representative plot of 3hi2one-G4 induced GIRK4 current decrease in response to optogenetic dephosphorylation by phosphatase 5-ptaseOCRL (20 μM 3hi2one-G4, *mauve open circle*). *C*, the decrease in current (%current remaining) is reduced when GIRK4 channels are studied in the presence of 3hi2one-G4. *D*, 5-ptaseOCRL-mediated decrease in GIRK4 current is characterized by monoexponential fits in the presence of 5-ptaseOCRL in the presence (*mauve circles*) and the absence of 3hi2one-G4 (*black circles*). *E*, 3hi2one-G4 increases the τ (tau) of current decrease, following activation of 5-ptaseOCRL. Data are currents recorded from HEK293T cells using patch clamp in the whole-cell mode and are shown as means ± SD for six to seven cells per group. Statistical significance was calculated using unpaired Student's *t* tests using GraphPad Prism (∗∗*p* < 0.005; ∗∗∗*p* < 0.0005). GIRK, G protein–sensitive inwardly rectifying potassium channel; HEK, human embryonic kidney cell line; 3hi2one, 3-[2-(3,4-dimethoxyphenyl)-2-oxoethyl]-3-hydroxy-1-(1-naphthylmethyl)-1,3-dihydro-2H-indol-2-one; PIP_2,_ phosphatidylinositol-4,5-bisphosphate.

To test whether 3hi2one-G4 activation of GIRK4 is PIP_2_ dependent, we utilized a light-activated 5′-phosphatase system that dephosphorylates [PI(4,5)P_2_] to PI(4)P (28). In this two-component system from plants, the prenylated CIBN (cryptochrome-interacting basic helix–loop–helix N-terminal fragment) transcription factor is localized in the plasma membrane, whereas the cryptochrome 2 (CRY2) photosensitive protein is activated to interact with CIBN and thus translocates to the cell surface upon blue-light exposure. A PIP_2_ lipid 5′-phosphatase is linked to the C-terminal end of CRY2 so that upon blue-light activation it is brought to the plasma membrane to dephosphorylate PI(4,5)P_2_ to PI(4)P. We conducted this experiment using whole-cell patch clamp (see the Experimental procedures section). Figure 3*A* shows a decrease in GIRK4 current upon blue-light stimulation, suggesting that PIP_2_ dephosphorylation by the 5′-phosphatase resulted in less remaining current. Comparison of the records of the same experiment but in the presence of 3hi2one-G4 decreased GIRK4 current inhibition and yielded a greater remaining current (Fig. 3*B*). Normalization of the basal currents prior to 5′-phosphatase activation to 1 showed clear differences not only in the remaining current but also in the relative kinetics of inhibition upon phosphatase activation (Fig. 3, *C*–*E*). Quantification of several cells indicated that 3hi2one-G4 stabilized interactions with PIP_2_ such that the 5′-phosphatase inhibited half the current (Fig. 3*D*) and with significantly slower kinetics (Fig. 3*E*). These results prompted us to explore further the binding site of 3hi2one-G4 and its relationship to the PIP_2_ binding site.

### The PI(4,5)P_2_ biosensor, iRFP-PHPLCδ1, translocates similarly into the cytoplasm upon depletion of the PI(4,5)P_2_ species by the 5PTASE_OCRL_, regardless of the presence of 3hi2one-G4

In order to exclude possible effects of 3hi2one-G4 on the 5′-phosphatase activity, we performed an additional control experiment. Using total internal reflection fluorescence (TIRF) microscopy, we monitored levels of iRFP-PHPLCδ1 at the cell surface. Following activation of CRY2-5ptaseOCRL, we observed significant depletion of PI(4,5)P_2_ under control as well as 3hi2one-G4–treated conditions, as indicated by the decrease in cell-surface fluorescence of iRFP-PHPLCδ1 (Fig. S2). These data show that iRFP-PHPLCδ1 redistributes away from the cell membrane at the same rate irrespective of the presence of 3hi2one-G4, indicating that 3hi2one-G4 does not alter the activity of the 5ptase_OCRL_. Thus, our data showing the strengthening of GIRK4–PIP_2_ interactions are due to the effect of 3hi2one-G4 on GIRK4 itself and not on the phosphatase, 5ptase_OCRL_.

### Modeling 3hi2one-G4 and GIRK4 channel interactions

**Figure 4:**
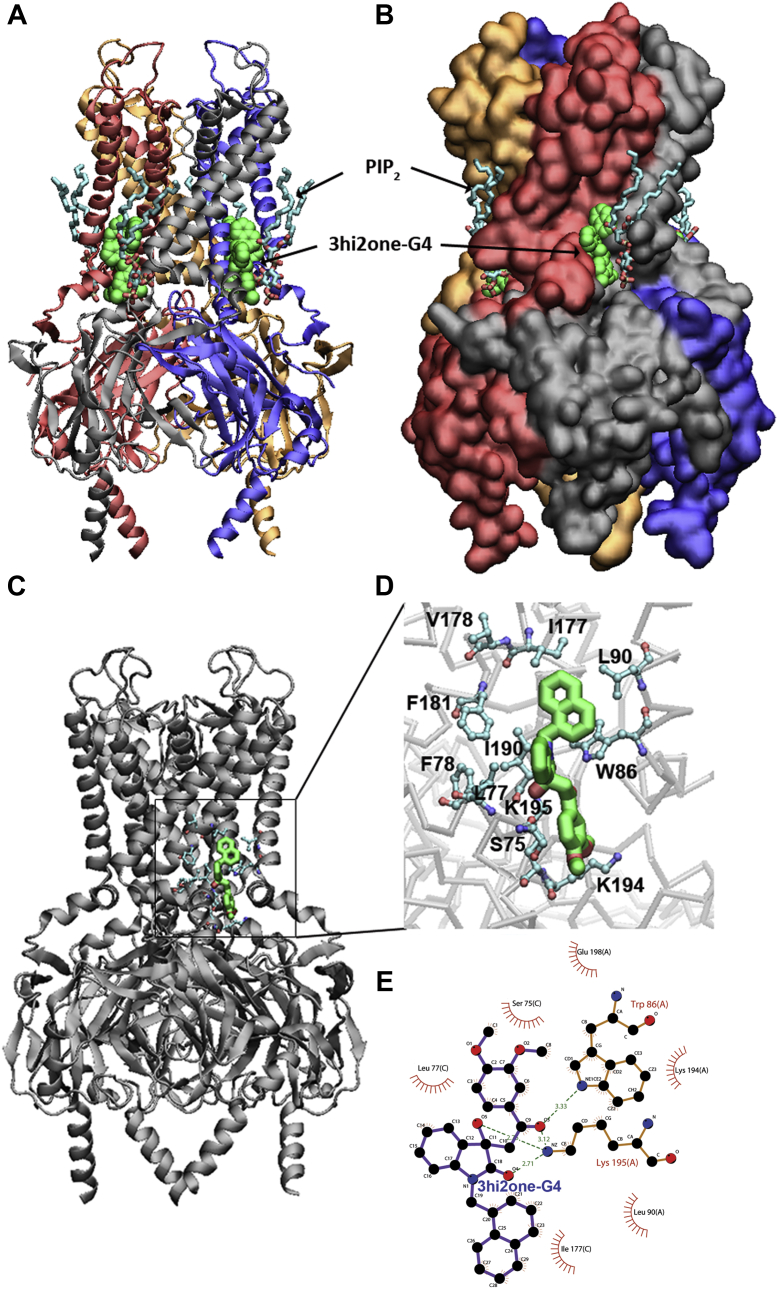
**Model of 3hi2one–GIRK4 channel complex and interactions between 3hi2one-G4 and GIRK4 channel.***A*, GIRK4 is rendered as NewCartoon; *B*, as molecular surface. 3hi2one molecules are rendered in van der Waals spheres colored in *green*, PIP_2_ molecules are rendered in *licorice* colored by atom types. *C*, molecular docking predicted 3hi2one-G4–GIRK4 channel complex. *D*, the critical interacting residues in 3hi2one-G4 binding site of GIKR4 channel. The 3hi2one-G4 is shown by *sticks* colored by atom types (C: *green*, N: *blue*, and O: *red*). *E*, 2D plot of the detailed interactions between 3hi2one-G4 and GIRK4 channel. Hydrogen bonds: K195 and W86; hydrophobic interactions: S75, L77, L90, I177, K194, and E198. GIRK, G protein–sensitive inwardly rectifying potassium channel; 3hi2one, 3-[2-(3,4-dimethoxyphenyl)-2-oxoethyl]-3-hydroxy-1-(1-naphthylmethyl)-1,3-dihydro-2H-indol-2-one; PIP_2_, phosphatidylinositol-4,5-bisphosphate.

The binding site of 3hi2one-G4 was predicted to be near the PIP_2_ binding site in the GIRK4 channel (Fig. 4, *A* and *B*), using molecular docking simulations (see the Experimental procedures section). These simulations depicted 3hi2one-G4 to interact with the GIRK4 through transmembrane domains 1 and 2 (TM1 and TM2) and the slide helix (S-HLX) (Fig. 4, *A* and *C*). It formed hydrogen-bond interactions with residues K195 and W86; van der Waals interactions with S75, L77, L90, I177, K194, and E198; pi–pi interactions with W86; and cation–pi interaction with K195 (Fig. 4, *D* and *E*).

### Mutations of the predicted 3hi2one-G4 binding site on GIRK4 channel

**Figure 5:**
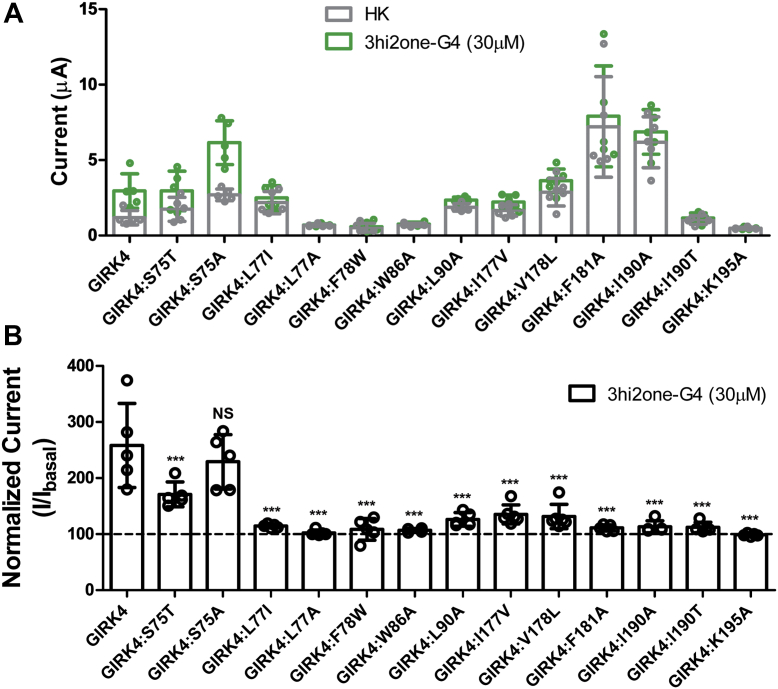
**Mutations of the predicted 3hi2one-G4 binding site on GIRK4 channel expressed in *Xenopus* oocytes.***A*, GIRK4 basal (HK, *gray bars*) and 3hi2one-G4 activator–induced current (*green bars*). *B*, normalized 3hi2one-G4 activator–induced current (I/I_basal_). The *asterisks* indicate significant differences of GIRK4 channel mutants by one-way ANOVA Tukey’s tests (∗∗∗*p* < 0.001) compared with GIRK4 wildtype channel (data are mean ± SD, N ≥ 4). GIRK, G protein–sensitive inwardly rectifying potassium channel; 3hi3one, 3-[2-(3,4-dimethoxyphenyl)-2-oxoethyl]-3-hydroxy-1-(1-naphthylmethyl)-1,3-dihydro-2H-indol-3-one; HK, high potassium.

To validate the 3hi2one-G4 binding site in the GIRK4 channel, we performed site-directed mutagenesis studies on predicted residues and characterized TEVC responses to 3hi2one-G4 of mutants relative to GIRK4 wildtype expressed in *Xenopus* oocytes. Figure 5*A* shows the raw data of the 3hi2one-G4 effects over basal currents. As some of the mutants affected the levels of basal currents, the stimulated mutant currents were normalized to their own basal currents (Fig. 5*B*). Mutations on all GIRK4 residues predicted to interact with 3hi2one-G4 impaired significantly the ability of the compound to stimulate significantly basal currents, with the exception of one. Mutation of residue S75 to Ala did not yield a significant change in the ability of 3hi2one-G4 to stimulate the GIRK4 mutant currents, whereas a Thr mutation significantly but partially affected the compound’s effectiveness. Of the remaining mutants, GIRK4:L77A, GIRK4:F78W, GIRK4:W86A, GIRK4:I190T, and GIRK4:K195A abolished 3hi2one-G4–induced channel activity. Mutants GIRK4:L77I, GIRK4:L90A, GIRK4:I177V, GIRK4:F181A, and GIRK4:I190A showed reduced 3hi2one-G4–induced currents. These data provided validation for the predicted 3hi2one-G4 binding site.

### 3hi2one-G4 activates GIRK4-mimetic GIRK2∗ channel mutants

**Figure 6:**
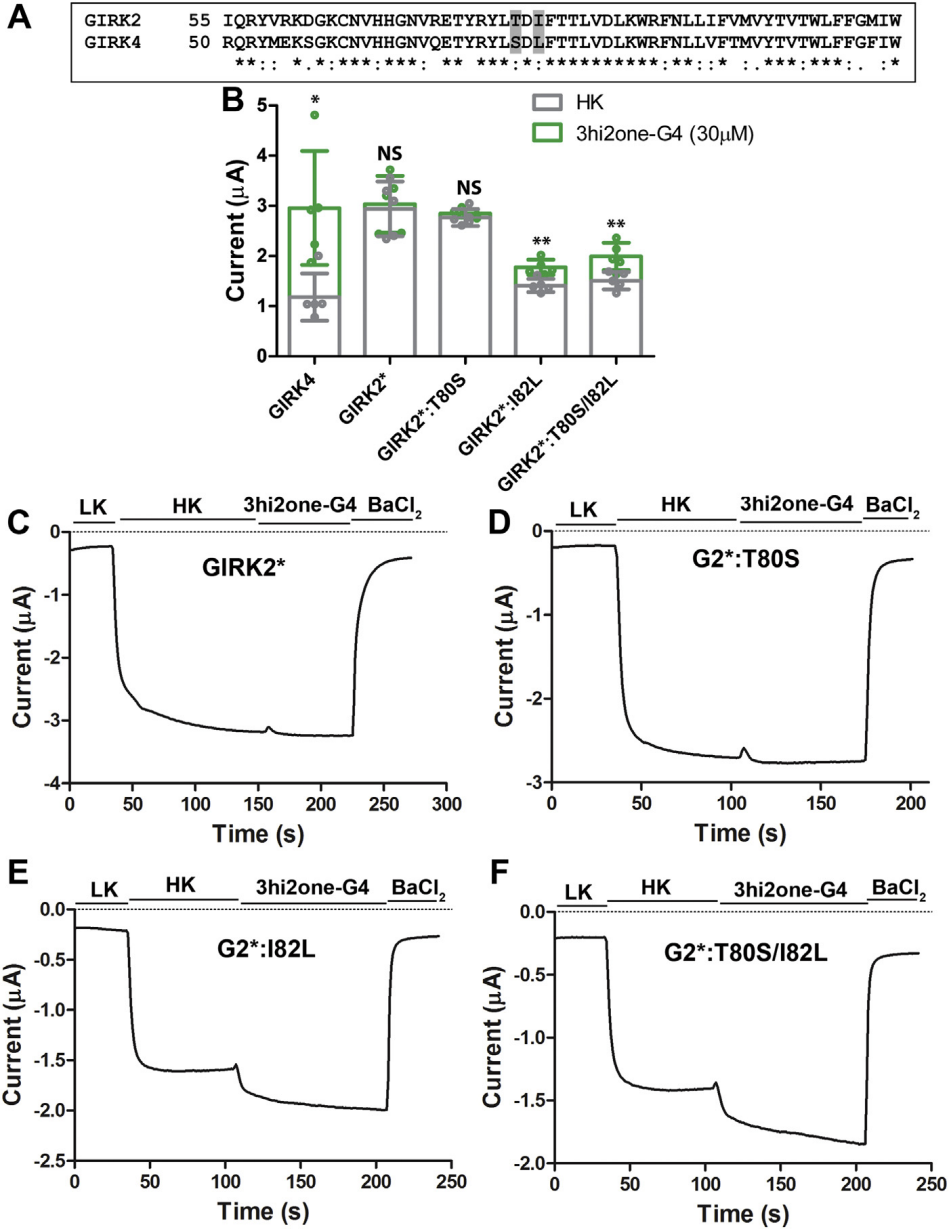
**3hi2one-G4 activates the GIRK2∗ channel mutants expressed in *Xenopus* oocytes.***A*, local sequence alignment between GIRK2 and GIRK4 near the slide helix region. Residues T80 and I82 in GIRK2; S75 and L77 in GIRK4 are highlighted in *gray* (∗: conserved, :: semiconserved, and .: similar residues). *B*, GIRK4, GIRK2∗, and mutant channels basal (HK, *gray bars*), and 3hi2one-G4 activator–induced current (*green bars*). The *asterisks* indicate significant differences between basal and 3hi2one-G4–induced currents by unpaired Student's *t* tests (∗∗*p* < 0.01, ∗*p* < 0.05) (data are mean ± SD, n = 5). *C*, representative traces of responses to the 3hi2one-G4 compound (30 μM) of GIRK2∗ channel. *D*, GIRK2∗:T80S. *E*, GIRK2∗:I82L. *F*, GIRK2∗:T80S/I82L. GIRK, G protein–sensitive inwardly rectifying potassium channel; HK, high potassium; 3hi2one, 3-[2-(3,4-dimethoxyphenyl)-2-oxoethyl]-3-hydroxy-1-(1-naphthylmethyl)-1,3-dihydro-2H-indol-2-one.

By comparing sequences between GIRK2 and GIRK4, there are only two slide helix residues different in the predicted 3hi2one-G4 binding site, T80 and I82 for GIRK2, and the corresponding S75 and L77 for GIRK4 channels (Fig. 6*A*). Therefore, these two residues could contribute to the selectivity of 3hi2one-GIRK4 for GIRK4 over GIRK2 channels. Since we found GIRK4:L77I, much more than GIRK4:S75T reduced the channel response to 3hi2one-G4 (Fig. 5*B*), we proceeded with the corresponding GIRK2 mutations: I82L and GIRK2∗:T80S, testing whether GIRK2∗ could be made sensitive to 3hi2one-G4. Strikingly, GIRK2∗:I82L and GIRK2∗:T80S/I82L but not GIRK2:T80S could be activated by 3hi2one-G4 at a concentration of 30 μM (Fig. 6). These results implicate the GIRK4:L77 as a selectivity critical residue for 3hi2one-G4 activity.

### Molecular dynamics simulations on 3hi2one-G4–GIRK4 channel complex

**Figure 7:**
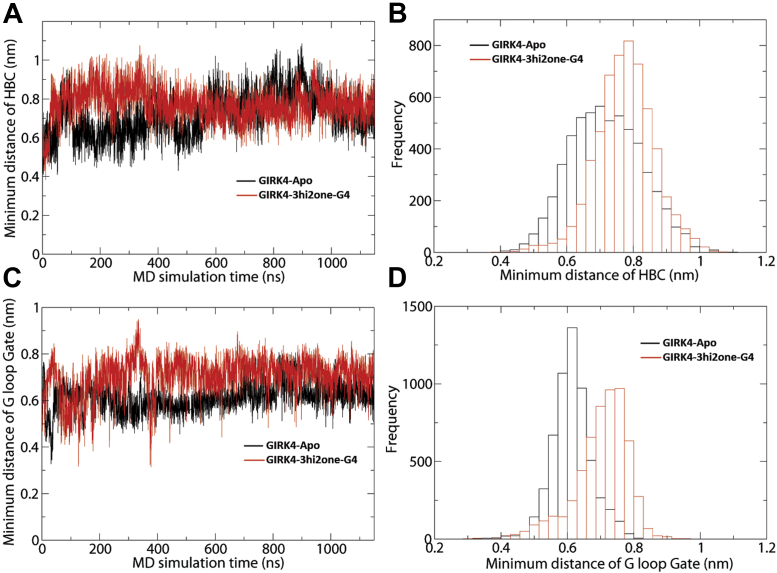
**3hi2one-G4 facilitates the opening of HBC and G-loop gates of GIRK4 channel.***A*, minimum distance of HBC gate as function of time. *B*, histogram plot. *C*, minimum distance of G-loop gate as function of time. *D*, histogram plot. GIRK, G protein–sensitive inwardly rectifying potassium channel; HBC, helix bundle crossing; 3hi2one, 3-[2-(3,4-dimethoxyphenyl)-2-oxoethyl]-3-hydroxy-1-(1-naphthylmethyl)-1,3-dihydro-2H-indol-2-one.

To further probe the molecular basis of the 3hi2one-G4 activation of GIRK4 homomeric channel, we performed 1 μs-long molecular dynamics (MD) simulations on the GIRK4 channel in the presence and absence of the 3hi2one-GIRK4 activator. Fig. S3 depicts the permeation pathway and the position of the two major channel gates, the HBC and G loop. Figure 7 shows the minimum distances of each of the two channel gates as a function of time from the Apo (without the ligand) GIRK4 and GIRK4/3hi2one-G4 MD simulation trajectories. 3hi2one-G4 facilitates the opening of HBC gate (7.8 Å *versus* 7.0 Å) and G-loop gate (7.2 Å *versus* 6.2 Å). In Fig. S3, we see a K^+^ ion pass through the GIRK4 channel (when bound to 3hi2one-G4 and PIP_2_) during the MD simulation. Starting at 1.012 μs, one K^+^ ion passed the HBC and G-loop gates within 52 ns.

To characterize the binding site residue interactions with 3hi2one-G4, we performed molecular mechanics–generalized born surface area binding free energy and per residue binding energy decomposition calculations. Table S1 shows the binding free energy contribution from each residue in the binding site. According to calculated free energies of interaction, the major interacting residues were F181 (−2.51 kcal/mol), L77 (−1.90 kcal/mol), W86 (−1.49 kcal/mol), K195 (−1.03 kcal/mol), and I190 (−0.91 kcal/mol). L77, the GIRK isoform selectivity contributing residue (Fig. 6), interacts with 3hi2one-G4 through van der Waals interactions. The minimum distance between residue L77 and 3hi2one-G4 plotted as a function of time was ≤3 Å throughout the simulation (Fig. S4*B*).

### 3hi2one-G4 activates the GIRK4 channel in a G protein–independent manner

**Figure 8:**
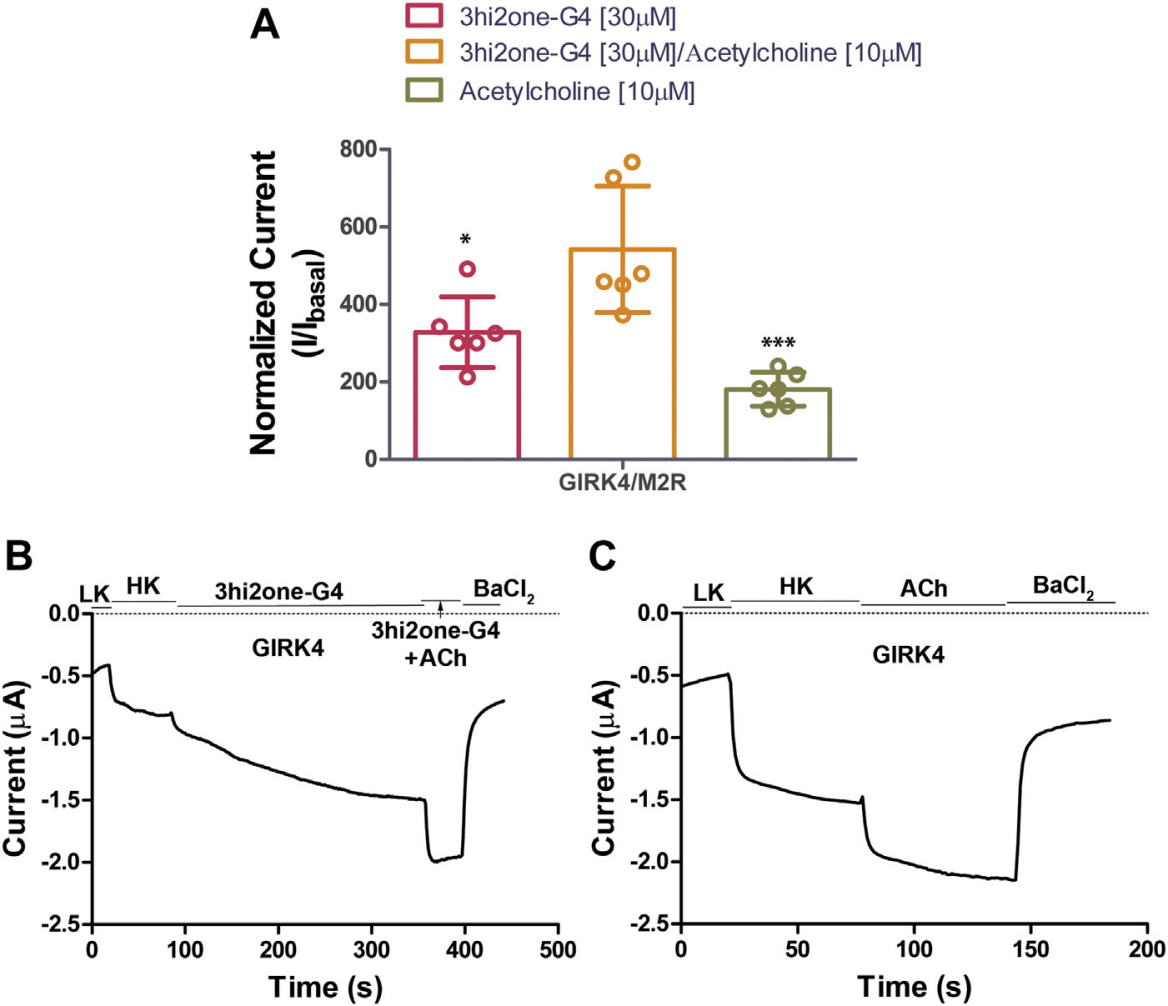
**3hi2one-G4 activates GIRK4 channel independent of G proteins.***A*, TEVC recordings of GIRK4 channel coexpressed with the M2R receptor in *Xenopus oocytes.* Activator (3hi2one-G4)/agonist (acetylcholine; ACh)-induced current (I/I_basal_) was normalized to HK current. The *asterisks* indicate significant differences tested by one-way ANOVA Tukey’s test (∗*p* < 0.05, ∗∗∗*p* < 0.001) (data are mean ± SD, N = 6). *B*, representative traces of responses of GIRK4 channel to the 3hi2one-G4 (30 μM)/ACh (10 μM). *C*, ACh (10 μM). GIRK, G protein–sensitive inwardly rectifying potassium channel; 3hi2one, 3-[2-(3,4-dimethoxyphenyl)-2-oxoethyl]-3-hydroxy-1-(1-naphthylmethyl)-1,3-dihydro-2H-indol-2-one; HK, high potassium; TEVC, two-electrode voltage clamp.

GIRK channels can be activated through G protein signaling, that is, directly by the Gβγ subunit through G protein–coupled receptor activation (9). To characterize whether 3hi2one-G4 activates GIRK4 channels in a G protein–dependent manner, we coexpressed the Gi-coupled M2R receptor, together with the GIRK4 channel in *Xenopus oocytes* for TEVC experiments. Figure 8 shows GIRK4 channel activation by the M2R agonist ACh (10 μM) and by 3hi2one-G4 (30 μM). Coapplication of the two activators showed an additive effect. This result is consistent with the notion that 3hi2one-G4 directly activates the GIRK4 channel in a manner independent of G protein signaling.

### 3hi2one-G4 activates aldosteronism disease–related GIRK4 channel mutants

**Figure 9:**
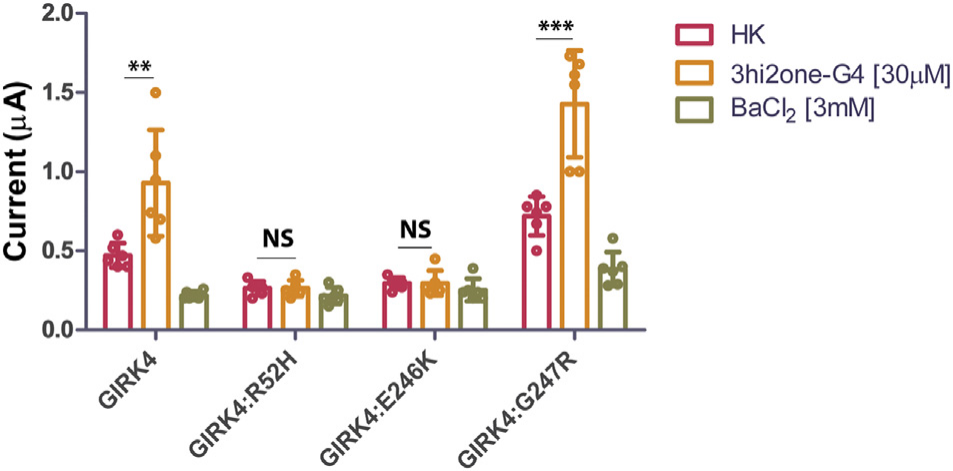
**The activity of 3hi2one-G4 on disease-related GIRK4 channel mutants.** TEVC recordings of GIRK4, GIRK4:R52H, GIRK4:E246K, and GIRK4:G247R channels expressed in *Xenopus oocytes*. The *asterisks* indicate significant differences tested by unpaired Student's *t* test (∗∗∗*p* < 0.001, ∗∗*p* < 0.01) HK basal current (data are mean ± SD, N = 6). GIRK, G protein–sensitive inwardly rectifying potassium channel; 3hi2one, 3-[2-(3,4-dimethoxyphenyl)-2-oxoethyl]-3-hydroxy-1-(1-naphthylmethyl)-1,3-dihydro-2H-indol-2-one; HK, high potassium; TEVC, two-electrode voltage clamp.

To test whether 3hi2one-G4 could activate GIRK4 channel mutants implicated in PA, GIRK4:R52H, GIRK4:E246K, and GIRK4:G247R, we performed TEVC experiments on these GIRK4 channel mutants expressed in *Xenopus* oocytes. Mutants GIRK4:R52H and GIRK4:E246K did not produce discernible Ba^2+^-sensitive currents above background, suggesting that these mutants are not functional. These two mutants also failed to be activated by 3hi2one-G4. In contrast, the mutant GIRK4:G247R, which is functional, was robustly activated by 3hi2one-G4 (Fig. 9).

### 3hi2one-G4 activates GIRK4 expressing in human adrenocortical 15 cells

**Figure 10:**
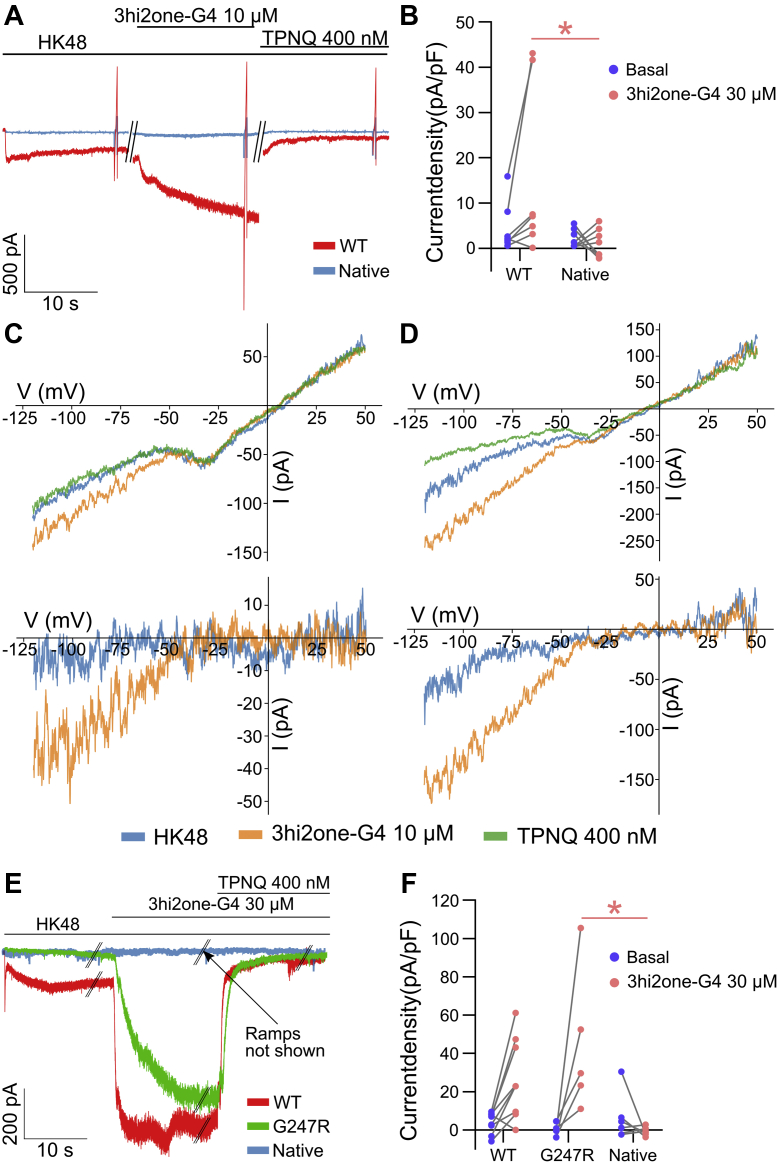
**The effect of 3hi2one-G4 on native and transfected HAC15 cells.***A*, representative traces from native and GIRK4:WT transfected HAC15 cells. *B*, current density of basal and 3hi2one-G4 response in native (mean ± SD: 2.09 ± 1.94 and 3.02 ± 2.27, respectively) and GIRK4:WT transfected (mean ± SD: 4.61 ± 5.548 and 19.95 ± 23.72, respectively) HAC15 cells shows that response to 3hi2one-G4 in transfected cells was larger compared with native cells. Voltage ramps (seen as brief bidirectional deflections in the current record) have been applied in each experiment (GIRK4:WT: n = 7 and native: n = 8). *C* and *D*, representative I–V relationships from native and GIRK4:WT transfected HAC15 cells, respectively. *Top*, representative raw I–V curves; *bottom*, net GIRK I–V relationships obtained by the subtraction of I–V curves recorded in the presence of TPNQ. *E*, representative traces from native, GIRK4:WT, and GIRK4:G247R transfected HAC15 cells. *F*, current density of basal and 3hi2one-G4 response in native (6.41 ± 12.27 and −0.76 ± 2.26, respectively), GIRK4:WT (4 ± 5.48 and 26.5 ± 20.21, respectively), and GIRK4:G247R (−0.52 ± 3.44 and 44.38 ± 37.34, respectively) transfected HAC15 cells shows that the response of GIRK4:G247R was significantly larger than the native but similar to GIRK4:WT (GIRK4:WT: n = 9, GIRK4:G247R: n = 5, and native: n = 6). GIRK, G protein–sensitive inwardly rectifying potassium channel; HAC15, human adrenocortical 15 cell line; 3hi2one, 3-[2-(3,4-dimethoxyphenyl)-2-oxoethyl]-3-hydroxy-1-(1-naphthylmethyl)-1,3-dihydro-2H-indol-2-one; TPNQ, Tertiapin-Q.

To assess the effect of 3hi2one-G4 in GIRK4-expressing cells, we measured whole-cell currents in human adrenocortical 15 (HAC15) cells (Fig. 10). HAC15 cells express GIRK4 channels endogenously; therefore, initially, we measured the response to 3hi2one-G4 in native and GIRK4:WT transfected cells (Fig. 10*A*). We found that 3hi2one-G4 activated inwardly rectifying GIRK currents, which were blocked by 400 nM Tertiapin-Q (Alomone Labs; catalog no.: STT-170; Figure 10, *C* and *D*). Basal current of native cells was similar to the transfected (Fig. 10*B*); however, 3hi2one-G4 evoked larger current in transfected cells compared with native (Fig. 10*F*). Next, we transfected HAC15 cells with GIRK4:G247R and tested the effect of 3hi2one-G4 (Fig. 10*E*). In this experiment, the batch of cells that was used had less endogenous GIRK4 current, showing no response to 3hi2one-G4. However, both GIRK4:WT and GIRK4:G247R transfected cells showed large 3hi2one-G4–evoked currents (Fig. 10*E*).

## Discussion

There is a limited number of GIRK channel modulators available currently, especially isoform-selective activators. ML297 was discovered through a high-throughput screen as the first urea scaffold GIRK1-selective potent activator, showing a ∼10-fold isoform selectivity in potency for activation of GIRK1/2 over GIRK1/4 channels (29). VU0810464 (30) was discovered as a nonurea GIRK1 activator, which shows comparable isoform selectivity to ML297 for GIRK1/2 over GIRK1/4 channels. Ivermectin (IVM), a Food and Drug Administration–approved antiparasitic drug, was also shown to be a GIRK channel activator, showing some selectivity for GIRK1/2 over GIRK1/4 channels (31). GAT1508 was discovered as a urea-scaffold ML297 analog, selectively activating the GIRK1/2 but not the GIRK1/4 channel (24). LOGO5 (the Light-Operated GIRK channel Opener) was discovered as the first photoswitchable GIRK channel opener selectively activating GIRK channels containing the GIRK1 subunit, such as GIRK1/2 and GIRK1/4 channels (32). GiGA-1 (33), another urea-based activator, was presented as a GIRK1/2 channel selective activator proposed to act on the alcohol-binding site. VU0529331, the first small-molecule activator of a homomeric GIRK channel, showed selectivity of activation not only for GIRK2 over GIRK4 but also for GIRK1/2 over GIRK1/4 channels (34). Even though the progress made toward discovery of GIRK channel modulators has been promising, a GIRK4 homomeric channel–specific activator had not been reported prior to the present report.

Here, we report the GIRK4 homomeric channel activator, 3hi2one-G4, which selectively stimulates homomeric GIRK4 and not homomeric GIRK2 or heteromeric GIRK1/2 or GIRK1/4 channel activity. 3hi2one-G4 activates the GIRK4 channel in a manner independent of G proteins. We characterized the potential binding site of 3hi2one-G4 in the GIRK4 homotetramer by using homology modeling, molecular docking, and MD simulations. The predicted binding site was validated by mutagenesis and electrophysiological studies. The unique predicted binding site is located near the PIP_2_ binding site. By comparing the sequences between the pockets of GIRK2 (insensitive to 3hi2one-G4) and GIRK4 (sensitive to 3hi2one-G4) channels, we show that residue L77 of GIRK4 is a major determinant of isoform selectivity for activation by 3hi2one-G4. The GIRK4:L77I mutant shows significantly less stimulation of its activity by 3hi2one-G4, whereas the corresponding GIRK2:I82L gains significant stimulation of its activity by the compound. IVM was reported to be a selective activator for GIRK1/2 over GIRK1/4 channels (31). By using chimeras and mutagenesis studies of GIRK2 and GIRK4 channels, I82 in GIRK2 (S-HLX) was identified as a major determinant for isoform specificity. GIRK2(I82) corresponds to GIRK4(L77). In addition, GIRK1/2(W91A) (TM1) and GIRK1/2(I195A) (TM2) were shown to reduce IVM-induced activation. These residues are located near the PIP_2_ binding site. Although the interactions between IVM and GIRK1/2 were not modeled, it appears that IVM may share a similar binding site for GIRK2 channel activation to 3hi2one-G4 and could also share a similar molecular mechanism of activation with the 3hi2one-G4 site in GIRK4. An Ile (or Val) residue is required in position 82 for IVM activation on GIRK1/2. In contrast, a Leu is required at position 77 for 3hi2one-G4 activation of GIRK4.

Our computational model shows that 3hi2one-G4 binds near PIP_2_, which is required for GIRK channel activation (12). The binding of 3hi2one-G4 enhances interactions between the channel and PIP_2_ and therefore enables channel opening (Fig. 3). The MD simulation results showed that the minimum distances of HBC and G-loop gates were increased in the GIRK4/PIP_2_/3hi2one-G4 system compared with the GIRK4/PIP_2_ system.

Residues K195 and W86 (corresponding to K200 and W91 in the GIRK2 channel) were predicted to be critical residues interacting with 3hi2one-G4 through hydrogen-bond and cation–pi interactions (Fig. 4*D*). Mutation of these residues to alanine abolished the 3hi2one-G4 stimulatory effect. K195 and W86 also interact with PIP_2_; therefore, mutations of these residues also affect basal activity of the channel (35). L77 was also predicted to be a critical interacting residue with 3hi2one-G4 through van der Waals interactions. Mutation of this residue to Ala or Ile (the GIRK2 corresponding residue) abolished or nearly abolished 3hi2one-G4–induced channel activity, respectively (Fig. 5*A*). In contrast, for another nonconserved residue S75 in GIRK4, corresponding to T80 in GIRK2, the mutant GIRK4:S75T had a milder but significant effect, whereas GIRK4:S75A did not affect 3hi2one-G4–induced channel activity. This supports the conclusion that L77 in GIRK4 is a selectivity critical residue of the channel for 3hi2one-G4. It is worth noting that mutants G4:F181A and G4:I190A produced eightfold and sevenfold basal current increases, respectively, compared with the wildtype channel. The role of these mutations in stimulating GIRK4 channel activity needs to be further investigated.

Three GIRK4 channel loss-of-function mutants were reported recently causing PA, GIRK4:R52H, GIRK4:E246K, and GIRK4:G247R (8). GIRK4 channel activators could serve as a potential strategy for treatment of patients with PA. The compound 3hi2one-G4 activates GIRK4:G247R but does not activate the completely nonfunctional mutants, GIRK4:R52H and GIRK4:E246K. This result is consistent with the effect of VU0529331 on the GIRK4:G247R mutant (8). Compared with VU0529331, which is not a completely selective homomeric GIRK channel activator, 3hi2one-G4 is a highly selective activator for GIRK4 homomeric channels, making it a better drug candidate for this disease. The present work suggests that pharmacological optimization efforts for 3hi2one-G4 are a worthy pursuit. In this regard, the limited brain distribution of GIRK4 in the brain (*e.g.*, POMC/VMN neurons) makes its peripheral use attractive in that appropriate chemistry could be engineered to prevent it from crossing the blood–brain barrier. Similarly, the low expression of GIRK4 homotetramers in the atria and pacemaking cardiac cells may prove not detrimental to activation by GIRK4-specific activators.

## Conclusions

In the present study, we identify 3hi2one-G4, the first isoform-selective activator of GIRK4 homomeric channels. This compound selectively activates GIRK4 homomeric channels, without activating GIRK1 heteromers (GIRK1/4 or GIRK1/2) or GIRK2 homomers. 3hi2one-G4 activates GIRK4 by binding at a site near the PIP_2_ binding site, which is formed by the TM1, TM2, and the S-HLX of the channel. The compound interacts with the channel through hydrogen bond, van der Waals, pi–pi, and cation–pi interactions. By comparing the sequences of the binding sites between GIRK4 and GIRK2, we identified L77 in GIRK4, as a major molecular determinant of isoform specificity. Mutation of the corresponding I82 to Leu in GIRK2 sensitized it to 3hi2one-G4 activation. MD simulation results showed that binding of 3hi2one-G4 facilitates the opening of GIRK4 channel gates. Based on the similarity of the binding site and activation mechanism with IVM, a previously identified activator of GIRK1/2 heteromeric channels, we suggest that 3hi2one-G4 and IVM share similar mechanisms of action for their corresponding targets. We conclude that 3hi2one-G4 could be a useful pharmaceutical probe to study GIRK4 channel function and to optimize for treatment of GIRK4-specific channelopathies, such as hyperaldosteronism.

## Experimental procedures

### TEVC

*Xenopus laevis* oocytes were isolated and microinjected as previously described (31). Human GIRK1 (pGEMSH vector), mouse GIRK2 (pXOOM vector), human GIRK4 (pGEMSH vector), and human GIRK14LK (GIRK4-10xGln-GIRK1, pXOOM vector) complementary DNA constructs were used for electrophysiology experiments. *In vitro* synthesized circular RNAs were injected in the amount of 2 to 5 ng per oocyte. Injected oocytes were incubated for 2 to 3 days at 18 ^°^C to allow for expression. Whole-cell oocyte currents were then measured using a GeneClamp 500 amplifier (Axon Instruments). Microelectrodes had resistances of 0.5 to 1 MΩ using a 3 M KCl solution in 1.4% agarose. Oocytes were perfused with a low K^+^ (LK) solution ND96 (2 mM KCl, 96 mM NaCl, 1 mM MgCl_2_, 1.8 mM CaCl_2_, 5 mM Hepes–Na) to establish a baseline for the recordings. Basal current was measured in a high potassium (HK) solution ND96K (96 mM KCl, 10 mM Hepes–K, 1 mM MgCl_2_, and 1.8 mM CaCl_2_). To block the current, the oocyte chamber was perfused with 3 mM BaCl_2_ in ND96K. Only the barium (Ba)-sensitive current was used for statistical analysis. To measure drug-induced activity of the channels, 30 μM 3hi2one-G4 in ND96K (HK) was used. Typically, oocytes were held at 0 mV (E_K_), and the current was monitored constantly using a ramp protocol with a command potential from −80 to +80 mV. Current amplitude was measured at the end of a sweep of 1 s. All currents were analyzed when they reached steady state. Error bars in the figure represent SEM (N > 4).

### Whole-cell patch clamp experiments

Whole-cell currents were recorded using an Axopatch 200B amplifier (Molecular Devices) using WinWCP software (University of Strathclyde). Currents were filtered through a lowpass Bessel filter at 2 kHz and were digitized at 10 kHz. Borosilicate glass electrodes were pulled using a vertical puller (Narishige) and had a resistance of 2 to 5 MΩ when filled with an intracellular buffer comprising 140 mM KCl, 2 mM MgCl_2_, 1 mM EGTA, 5 mM Na_2_ATP, 0.1 mM Na_2_GTP, and 5 mM Hepes buffered to pH 7.4 using KOH. Electrophysiology experiments were performed at room temperature. Cell capacitances ranged from 8 to 15 pF. Cells were cotransfected with 2 μg GIRK4 for the experiments in Figure 1*D* and with 2 μg GIRK4, 0.75 μg CIBN-GFP-CAAX, and 0.75 μg CRY2-5′-ptaseOCRL for the experiments in Figure 3, using polyethylenimine. The cells were maintained in Dulbecco's modified Eagle's medium supplemented with 10% fetal bovine serum and 1% penicillin and streptomycin at 37 °C in a 5% CO_2_ humidified atmosphere and were studied 48 h after transfection. GFP-expressing cells were selected for analysis using a Nikon epifluorescence microscope. Cells were held at 0 mV, and currents were recorded using a repeating ramp protocol from −80 mV to +80 mV in whole-cell mode. Cells were perfused directly using a multibarrel gravity–driven perfusion apparatus. Initially, in a physiological buffer containing 135 mM NaCl, 5 mM KCl, 1.2 mM MgCl_2_, 1.5 mM CaCl_2_, 8 mM glucose, and 10 mM Hepes; pH 7.4, GFP+ cells formed a giga-Ω seal with the patch pipette. Slight suction was applied to the cells to enter the whole-cell mode. GIRK channel activity was measured at −80 mV after transitioning to an HK buffer comprised of 5 mM NaCl, 135 mM KCl, 1.2 mM MgCl_2_, 1.5 mM CaCl_2_, 8 mM glucose, and 10 mM Hepes; pH 7.4. Currents were blocked using 5 mM Ba in HK.

### Light-activated phosphatase experiments

The light-activated phosphatase system to dephosphorylate PIP_2_ (36) is comprised of two fusion proteins: mCherry-CRY2-5′-ptase_OCRL_ and CIBN-GFP-CAAX. The 5′-ptase_OCRL_ system was activated using a 460 nm LED (Luminus) that was focused on the cells through the objective lens of an inverted microscope (Nikon). Cells were studied in whole-cell mode, and basal GIRK activity was assessed in HK. After currents in HK stabilized, cells were illuminated with blue light with an LED at 5 mW to activate the phosphatase. Current depletion after phosphatase activation was allowed to stabilize before current was blocked with 5 mM Ba^2+^ in HK.

Data for percent inhibition were calculated using Ba^2+^-sensitive currents:

$$\left[\frac{\text{Current after ptase activation}}{\text{Current in HK}}\right]\text{x }100$$

Currents were fitted to a monoexponential equation to extract τ values to measure the kinetics of current inhibition.

### TIRF microscopy

Cells seeded on 35 mm glass-bottomed petri dishes were transfected using polyethylenimine (1:4) with 1 μg of the near-infrared PI(4,5)P_2_ biosensor iRFP-PHPLCδ1, 0.75 μg each of CRY2-5PTASE_OCRL_ and CIBN-CAAX 48 h before analysis. Cells were studied in a solution comprised of (in millimolar): NaCl 130, KCl 4, MgCl_2_ 1.2, CaCl_2_ 2, Hepes 10, and pH was adjusted to 7.4 with NaOH. Blue-light activation was performed using a 445-nm laser, and iRFP was excited by a 647-nm laser (Coherent). Cells were first illuminated with 647λ to collect baseline images in TIRF mode. The CRY2-5ptase_OCLR_ system was activated for 10 s using 445 λ in epifluorescence mode. Postillumination images were collected in TIRF mode. The beams were conditioned for coherence with custom-built Keplerian beam expanders upstream of laser cleanup filters. Laser lines were tuned to provide 10 mW of incident light on a micro mirror positioned below a high numerical aperture apochromat objective (60×, 1.5 numerical aperture; Olympus) mounted on an RM21 microscope frame equipped with a piezo-driven nanopositioning stage (MadCity Labs). iRFP was imaged through a 665-nm longpass filter using a back-illuminated sCMOS camera (Teledyne Photometrics) controlled by Micro-Manager freeware (University of California San Francisco). All filters and mirrors were from Chroma. Lenses, pinholes, and diaphragms were from ThorLabs. Tetraspec beads (Thermo Fisher Scientific) were routinely imaged to map the sCMOS chip and to calibrate the evanescent field depth to 100 nm. Images were captured at 5 s intervals with a 10 ms exposure time to minimize photobleaching, and the data were saved as stacked TIFF files. Image analysis was performed with ImageJ (National Institute of Mental Health).

### HAC15 cell culture, transfection, and electrophysiology

Cells were acquired from American Type Culture Collection (catalog no.: CRL3301) and cultured as described (37). Cells were grown in Dulbecco’s modified Eagle’s/Ham’s F-12 medium (Gibco; catalog no.: 11330-05) containing 10% Cosmic calf serum (Hyclone; catalog no.: SH30), 1% l-glutamine (Sigma–Aldrich; catalog no.: A7506), 1% ITS (Becton Dickinson; catalog no.: FAL354352), and penicillin–streptomycin (Sigma–Aldrich; catalog no.: P4333).

For electrophysiological experiments, cells were transfected with Lipofectamine 3000 (Thermo Fisher Scientific) during passaging and plated on poly-l-lysine (Merck; catalog no.: P2636)–coated 13 mm cover slips. DNAs used for transfection in nanogram: GFP 0.1 and GIRK4:WT 0.4. Electrophysiological measurements were performed in the following 24 to 72 h.

Whole-cell currents were measured using Axopatch 200B (Molecular Devices). Holding potential was −80 mV, and voltage ramps of 400 ms were from −120 to +50 mV. No correction for junction potential (∼13 mV) was made. Cells were constantly perfused with LK solution (in millimolar): NaCl 136, KCl 4, CaCl_2_ 2, MgCl_2_ 2, Hepes 10, NaH_2_PO_4_ 0.33, glucose 10, and pH 7.4. Currents were recorded in HK (HK48) solution (in millimolar): NaCl 92, KCl 48, CaCl_2_ 2, MgCl_2_ 2, Hepes 10, NaH_2_PO_4_ 0.33, glucose 10, and pH 7.4. About 10 or 30 μM 3hi2one-G4 was used to induce channel activation. Electrode solution contained (in millimolar): NaCl 6, KCl 22, K-gluconate 110, MgCl_2_ 2, Hepes 10, EGTA–HOH 1, ATP-K_2_ 2, GTP–Tris 0.5, and pH 7.2. To get net GIRK current, 400 nM of the blocker Tertiapin-Q was added to the HK48 solution.

### Molecular modeling and simulations

#### Homology modeling

The GIRK4 channel model structure was based on a crystal structure of GIRK2 channel (Protein Data Bank ID: 3SYA). A sequence alignment between the GIRK4 and GIRK2 channels was generated by the ClustalW server (http://www.genome.jp/-tools/clustalw/). The GIRK2 channel shares high sequence identity with GIRK4 (81.4%) channels, which is much higher than the generally acceptable 30% sequence identity for homology modeling, and makes the GIRK2 crystal structure an excellent template for developing an accurate homology model for the GIRK4 channel. We used the MODELLER program (University of California, San Francisco) (38) to generate 10 initial homology models for GIRK4 based on the GIRK2 structural template (18) and selected the one with the best internal DOPE score from the program for modeling the compound and channel interactions.

#### Molecular docking

The GIRK4 channel model structure was prepared by the Protein Preparation Wizard module of Maestro (2017-1) program (Schrödinger, Inc). The binding site of the GIRK4 channel was defined by its proximity to the PIP_2_ head group. A grid box of 30Å × 30 Å × 30 Å was generated using the Receptor Grid generation module. The default parameters were used for grid generation. Flexible ligand dockings were performed using standard precision by the Glide program (Schrödinger, LLC) (39). The docked pose with the lowest standard precision score was selected and used as a reference ligand for induced-fit docking simulations (40). Default parameters were used for induced-fit docking simulations. The residues within 5 Å of ligand poses were selected for side-chain optimization by prime refinement. The XP scores were used for ranking of the ligand poses, and top five poses of docked ligand were saved for visual inspection and selection. The pose of docked 3hi2one-G4 with the lowest docking XP score was selected for MD simulations.

#### MD simulations

The GIRK4/PIP_2_ and GIRK4/PIP_2_/3hi2one-G4 structures were immersed in an explicit lipid bilayer of 1-palmitoyl-2-oleoyl-*sn*-glycero-3-phosphocholine, 1-palmatoyl-2-oleoyl-*sn*-glycero-3-phosphoethanolamine, 1-palmitoyl-2-oleoyl-*sn*-glycero-3-phospho-l-serine, and cholesterol at a molecular ratio of 25:5:5:1 (41) and a water box in 129.7 Å × 130.2 Å × 160.1 Å dimension by using the CHARMM-GUI Membrane Builder webserver (http://www.charmm-gui.org/?doc=input/membrane). About 150 mM KCl and extra neutralizing counter ions were added into the systems. The total number of atoms of the two systems were 164,403 and 164,643 for the GIRK4/PIP_2_ and GIRK4/PIP_2_/3hi2one-G4, respectively. The Antechamber module of AmberTools was used to generate the parameters for 3hi2one-G4 using the general AMBER force field. The partial charges for the 3hi2one-G4 were calculated using RESP restrained electrostatic potential charge-fitting scheme by *ab initio* quantum chemistry at the HF/6-31G∗ level (GAUSSIAN 16 program, Gaussian, Inc) (42). The PMEMD.CUDA program in AMBER16 (University of California, San Francisco) was used to conduct the MD simulations. The MD simulations were performed with periodic boundary conditions to produce isothermal–isobaric ensembles. An external voltage of 0.06 V/nm was added to the systems from the extracellular to the intracellular side (17, 43, 44). Long-range electrostatics were calculated using the particle-mesh Ewald method (45) with a 10 Å cutoff. Prior to production runs, energy minimization of the system was carried out. Subsequently, the system was heated from 0 to 303 K using Langevin dynamics with the collision frequency of 1 ps^−1^. During the heating, the GIRK4 channel was position-restrained using an initial constant force of 500 kcal/mol/Å^2^ and weakened to 10 kcal/mol/Å^2^, allowing lipid and water molecules to move freely. Then, the system went through 5 ns equilibrium MD simulations. Finally, a total of 1.2μs production MD simulation was conducted, and coordinates were saved every 100 ps for analysis. In total, 12,000 snapshot structures were collected during the MD simulations. The minimum distances between atoms in the trajectories were analyzed using the built-in utility of the GROMACS program (Groningen University).

## Data availability

All data are contained within the article.

## Supporting information

This article contains supporting information.

## Conflict of interest

The authors declare that they have no conflicts of interest with the contents of this article.
