# Supplementary material for: A novel small-molecule selective activator of homomeric GIRK4 channels
Source: J Biol Chem. 2022 May 4;298(6):102009. doi: 10.1016/j.jbc.2022.102009 (PMC9194863; doi:10.1016/j.jbc.2022.102009)
Supplement: Supplemental Figures S1–S4 and Table S1 [file mmc1.docx]

**A novel small molecule selective activator of homomeric GIRK4 channels**

**Supplementary materials**

Meng Cui^1,4^* Keman Xu^1^, Kirin D. Gada^1^, Boris Shalomov^2^, Michelle Ban^1^, Giasemi C. Eptaminitaki^1^, Takeharu Kawano^1^, Leigh D. Plant^1,4^, Nathan Dascal^2^, and Diomedes E. Logothetis^1,3,4^*

^1^ Department of Pharmaceutical Sciences, School of Pharmacy, Bouvé College of Health Sciences, Northeastern University, Boston, Massachusetts, 02115, USA

^2^ Department of Physiology and Pharmacology and Sagol School of Neuroscience, School of Medicine, Tel Aviv University, Tel Aviv, 69978, Israel

^3^ Chemistry and Chemical Biology, College of Science, Northeastern University, Boston, MA 02115, USA

^4^ Center for Drug Discovery, Northeastern University, Boston, MA 02115, USA

* To whom correspondence should be addressed: m.cui@northeastern.edu (MC); d.logothetis@northeastern.edu (DEL)


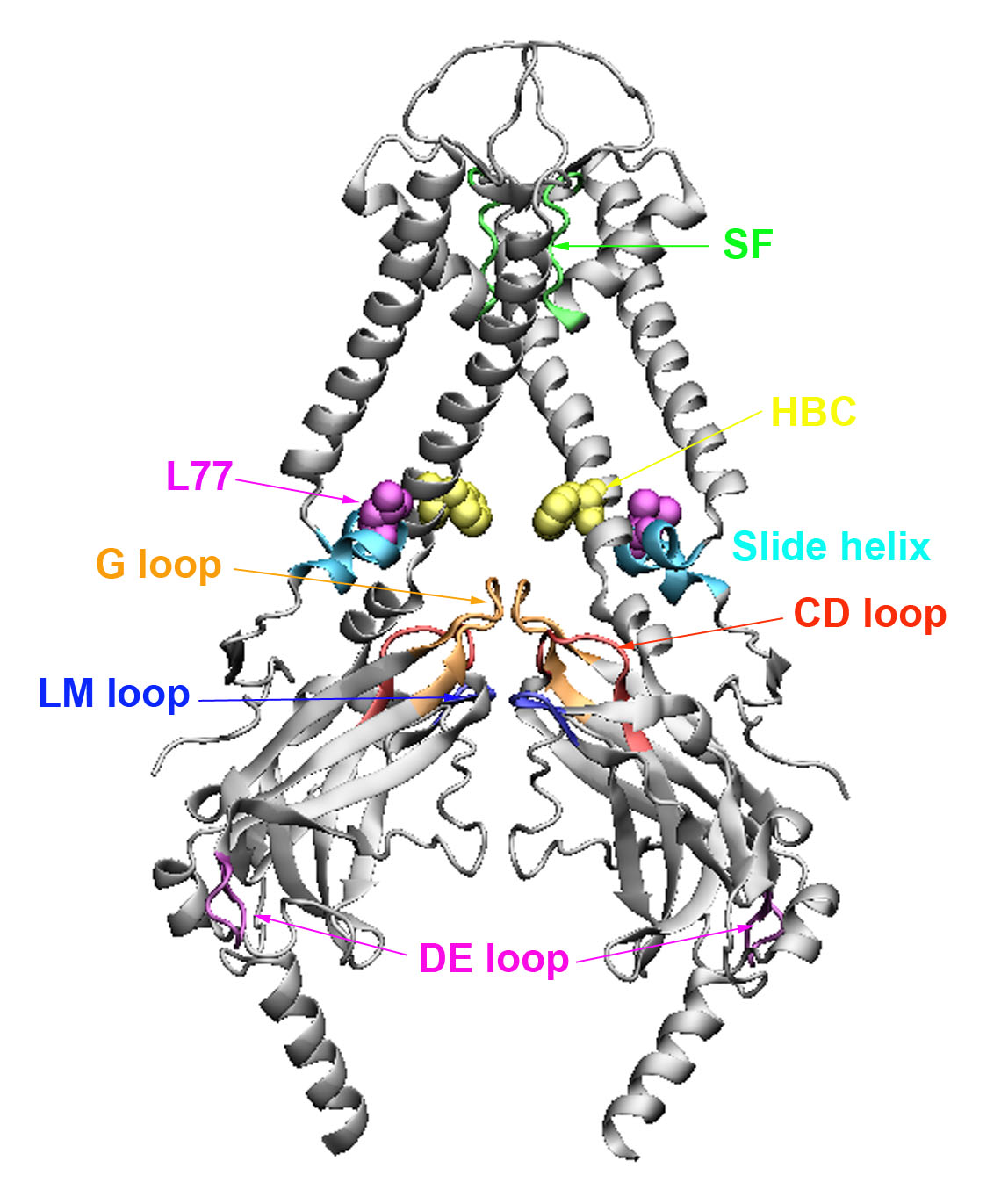


**Figure S1. GIRK4 channel model structure.** SF: selectivity filter; HBC: Helix Bundle Crossing (F187 in GIRK4); three critical loops: CD loop binds Na^+^ and is directly involved in the G loop-gating mechanism, DE and LM loops from adjacent subunits make up a cleft that comprises the binding site for a Gβγ dimer; Slide helix is a critical helix parallel to the plane of the plasma membrane that orchestrates interactions of PIP_2_ with the gates as well as of critical structural elements (e.g. CD loop, the critical L77 specificity residue for 3hi2one-G4 identified in this study) during gating.


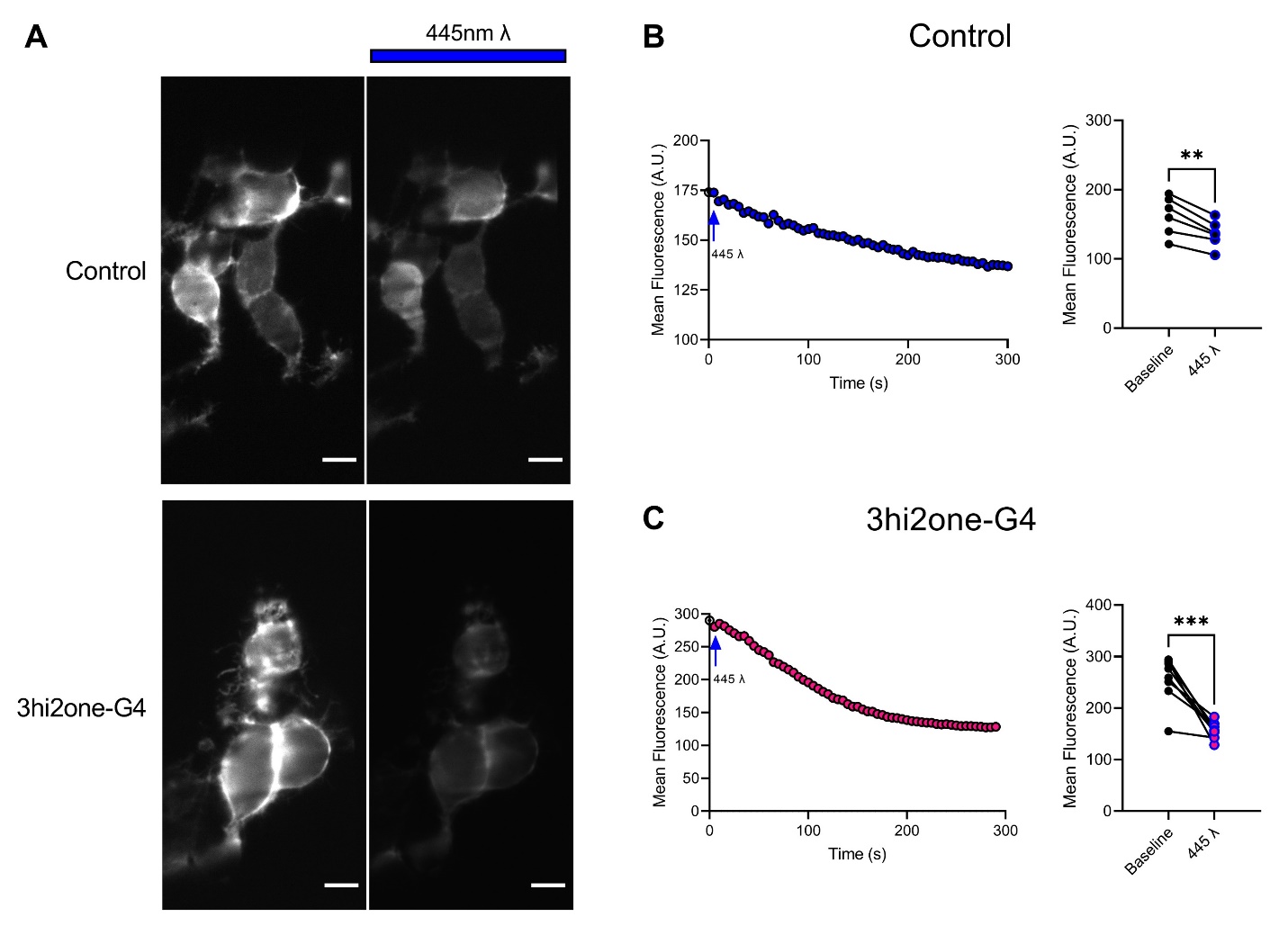


**Figure S2. 3hi2one-G4 does not affect the activity of the PIP_2_ phosphatase, CRY2-5ptaseOCRL.** 0.75µg of CRY2-5PtaseOCRL was co-expressed with 0.75µg CIBN-CAAX and 1µg of the near-infrared PI(4,5)P_2_ biosensor iRFP-PHPLCδ1 in HEK293-T cells. (A) Representative TIRFM images showing the surface fluorescence of cells transfected with CRY2-5PtaseOCRL and CIBN-CAAX before and after illumination with a blue laser, scale bar = 10µm. (B), A time course of cell surface fluorescence shows a decrease in iRFP fluorescence at the cell membrane after cells are illuminated with blue light where ‘0’ indicated the baseline timepoint; blue arrows indicate the initiation of illumination by blue light. (C). Summary data showing the surface fluorescence of cells before and after illumination with a blue laser in control and 3hi2one-G4 treated cells (summary data are mean fluorescence ± S.D. for 6-7 cells per experiment; p-values **p<0.005, ***p<0.0005, calculated using Student’s paired t-test in Graphpad Prism).


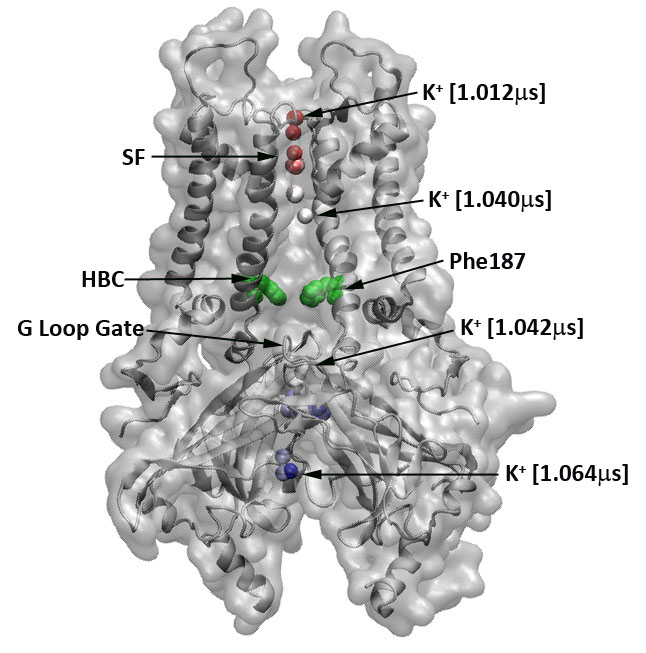


**Figure S3.** **Permeation of potassium ions through the GIRK4 channel gated by 3hi2one-G4.**


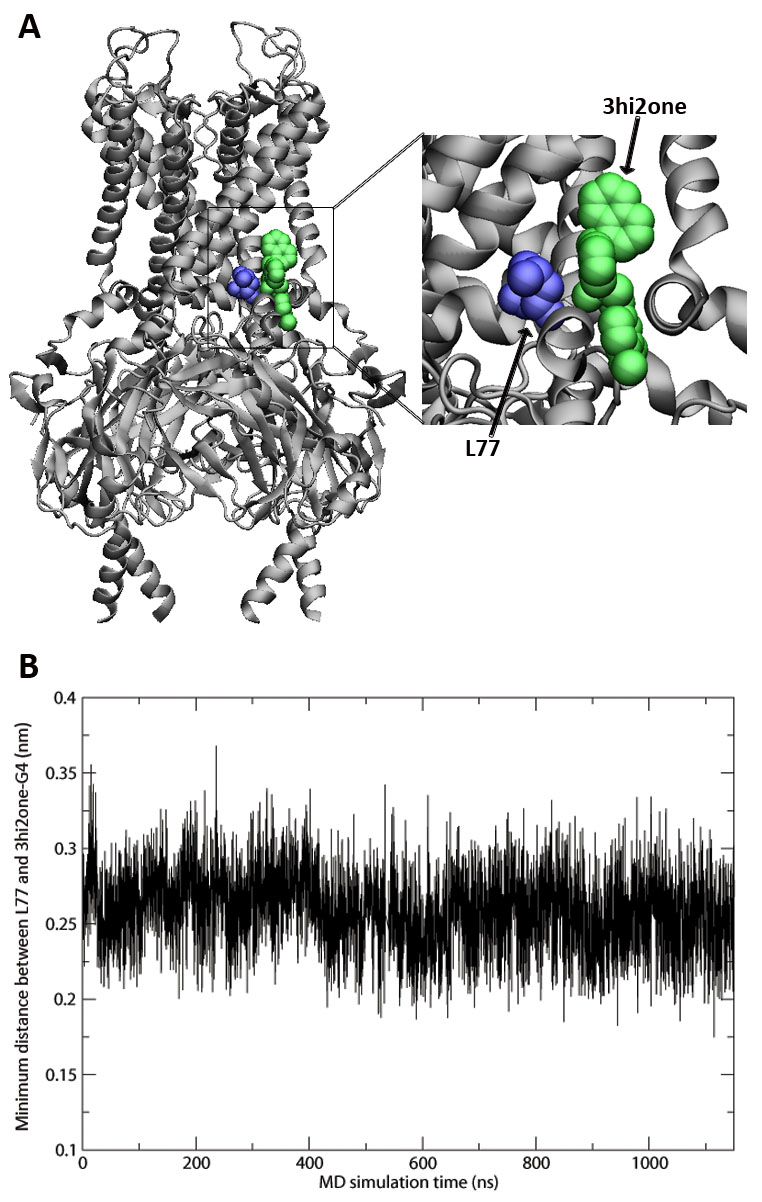


**Figure S4. Interactions between the Leu77 and 3hi2one-G4.** A. 3hi2one-G4 binding site in the GIRK4 channel, which shows residue L77 closely interacting with the compound. B. The minimum distance between residue L77 and 3hi2one-G4 as a function of time during the MD simulations.

**Table S1: Predicted critical residues of GIRK4 channel interacting with 3hi2one-G4 from MMGBSA binding free energy calculations.**

| **Residue** | **Interaction energy (Kcal/mol)** |
| --- | --- |
| F181 | -2.51 |
| L77 | -1.90 |
| W86 | -1.49 |
| K195 | -1.03 |
| I190 | -0.91 |
| S75 | -0.79 |
| D76 | -0.78 |
| L74 | -0.48 |
| V178 | -0.46 |
| I177 | -0.39 |
| F78 | -0.34 |
| K194 | -0.3 |
| L90 | -0.1 |
